# The birthplace and relative age effects in Brazilian olympic athletes: a cross-national comparison

**DOI:** 10.3389/fpsyg.2023.1135471

**Published:** 2023-07-10

**Authors:** Mabliny Thuany, Douglas Vieira, Marcos Lima, Jaíne Taniele Cavalcante, Tatiana Alcântara, Pantelis T. Nikolaidis, Beat Knechtle, Katja Weiss, Thayse Natacha Gomes

**Affiliations:** ^1^Centre of Research, Education, Innovation and Intervention in Sport (CIFI2D), Faculty of Sport, University of Porto, Porto, Portugal; ^2^Post-Graduation Program of Physical Education, Department of Physical Education, Federal University of Sergipe, São Cristóvão, Sergipe, Brazil; ^3^Department of Physical Education, Federal University of Sergipe, São Cristóvão, Sergipe, Brazil; ^4^School of Health and Caring Sciences, University of West Attica, Athens, Greece; ^5^Medbase St. Gallen Am Vadianplatz, St. Gallen, Switzerland; ^6^Institute of Primary Care, University of Zurich, Zurich, Switzerland

**Keywords:** birthplace effect, relative age effect, olympic games, athletes, country

## Abstract

**Purpose:**

Our purpose was to verify the existence of birthplace and relative age effect (RAE), as well as the association between birthplace effect and RAE among Brazilian athletes competing in the Olympic Games.

**Methods:**

Data included information about Brazilian Summer Olympic athletes from 1920 to 2021. To investigate RAE, athletes’ birthdate was distributed into birth quartiles (Q1: Jan–Mar; Q2: Apr–Jun; Q3: Jul–Sep; Q4: Oct–Dec), while birthplace effect was assessed considering the state and the region (Southeast; Northeast; South; North; and Midwest) of birth. The Chi-square test (χ^2^) was used to verify differences between the birthplace effect and RAE.

**Results:**

The sample consisted of 388 Brazilian athletes of both sexes, distributed in 38 sports modalities from 23 Brazilian states (Southeast = 66.5%; South = 14.4%; Northeast = 12.1%; North = 1.5%; Midwest = 5.4%). For both sexes, most of the athletes were from the São Paulo state (37.4%), followed by Rio de Janeiro (18.3%), both from the Southeast region. For birthdate distribution according to birthplace, it was observed that the North region presented the highest frequency of athletes born in Q1 (50%), followed by the Northeast and Southeast regions. No significant differences were found for the birthplace effect (χ^2^ = 5.69, value of *p* = 0.128) and RAE between sexes (χ^2^ = 0.530, value of *p* = 0.912), nor was shown an association between the birthplace effect and RAE.

**Conclusion:**

Most Brazilian Olympic athletes are from the Southeast region, but no RAE was established regarding their birthplace. Results from the present study can guide sports public policies in Brazilian regions, especially in the Midwest, North, and Northeast regions, which are underrepresented in Brazilian high-performance sports.

## 1. Introduction

The interest in understanding the role of contextual variables in athletes’ development and performance has increased in the last few years (Yan et al., 2016). The birthplace effect is the term used to characterize the role played by the place where an individual was born and grew up during his early development to their success (Cote et al., 2006; Hancock et al., 2018). The birthplace effect has been associated with the likelihood of an athlete achieving high levels of proficiency in sports (Rossing et al., 2018; Smith and Weir, 2020). Previous studies have highlighted the birthplace effect in different contexts (Smith and Weir, 2020; Leite et al., 2021), such as soccer (Teoldo and Cardoso, 2021), the Olympic Summer Games (Tozetto et al., 2017), and basketball (Faria et al., 2021). However, only few studies investigated the impact of the birthplace among Brazilian Olympic athletes over the years and, nonetheless the relevance, most of them are time-limited (Tozetto et al., 2017; Thuany et al., 2021b).

When the birthplace effect was studied in the Brazilian context, results present some differences. For example, studying athletes competing in Rio de Janeiro Olympic Games 2016, Tozetto et al. (2017), showed that most of the Brazilian athletes were from the Southeast and South regions. Further, among those competing in Tokyo Olympic Games 2020, athletes from the Southeast and Northeast regions presented a higher chance of being a medalist (Thuany et al., 2021b). Notwithstanding the Southeast and South regions present the best economic index among Brazilian regions (OECD, 2011), higher participation of athletes from the Northeast region can be related to public policy programs and the legacy of the Rio de Janeiro 2016 Summer Olympics, which lead to the development of the Northeast Olympic Training Center (*Centro de Formação Olímpica do Nordeste*; Fortaleza—Ceará) to support 26 Olympics sports disciplines (Ceará, 2017).

In addition to the birthplace effect, the relative age effect (RAE) has also been associated with the likelihood to become an elite athlete in different sports disciplines (Brazo-Sayavera et al., 2018; Gil et al., 2021). In summary, the RAE refers to the association between performance and the calendar month in which an athlete has been born (Roberts et al., 2021). If RAE is documented in a sport, the athletes born in given months outnumber those born in the other months (the comparisons are usually made between the first versus last months). This phenomenon explains that “early-born” athletes—at the beginning of their athletic career—are older than their “late-born” peers (Gil et al., 2021). An extreme example is that an athlete born on 1st January is almost 1 year older than an athlete born on 31st December of the same calendar year, though both compete in the same age group. Such age difference during the stage of sports development may provide an advantage to “early-born” athletes who usually outscore their peers in terms of anthropometric and physiological characteristics, which in turn reinforces their psychological characteristics and a mentality of a “winner” (Drenowatz et al., 2021; van Aalst and van Tubergen, 2021).

Considering the differences between the Brazilian macro-regions, it is expected a different distribution for both birthplace and RAE phenomena. For example, despite the Southeast region being pointed out as the economic center of the country, the region presents the best indicators of human development and is the most populous in the country. More people competing for specific positions within clubs and sports programs can create a competitive atmosphere among the states from this region. In addition, this selective pressure can be associated with the feeling of “victory at all costs,” and also reflect in the selection of the athletes, or the odds to provide resources for specific groups. No information is available about the connection between the birthplace effect and RAE in Brazilian Olympic athletes. The following research questions were put forward: (1) What is the birthdate distribution of the Brazilian Olympic athletes according to birthplace? and (2) What are the associations between birthplace and RAE in Brazilian athletes competing in the Olympic Games?

## 2. Methods

### 2.1. Design and sample

This is a retrospective study where secondary data from the official website of the Brazilian Olympic Committee^1^ was used. Considering missing information (e.g., no information about the place of residence or birthdate), as well as the exclusion of foreign athletes, the sample size was composed of 388 Brazilian athletes of both sexes (33.8% women), competing in the Summer Olympic Games editions from 1920 to 2020/21. Participants were distributed in 38 sports modalities, and they were born in 23 Brazilian states (Southeast = 66.5%; South = 14.4%; Northeast = 12.1%; North = 1.5%; and Midwest = 5.4%). Ethical review and approval was not required for the study on human participants in accordance with the local legislation and institutional requirements. Written informed consent from the participants was not required to participate in this study in accordance with the national legislation and the institutional requirements.

### 2.2. Data collection procedures

We downloaded all the data from the official website of the Brazilian Olympic Committee for athletes competing in Summer Olympic Games (1920 to 2020/21). Data comprised information about sex (female; male), birthdate (mm/dd/yyyy), birthplace (city, state), year of the Olympic Games, medals (gold, silver, bronze, or none), and/or the ranking position (i.e., classification in the competition), and sports modality participation. Sports modalities were categorized into three groups, i.e., team sports, individual sports, and mixed sports (when the competition can be performed individually and/or in teams, such as gymnastics, tennis, and badminton).

### 2.3. Birthplace effect and relative age effect

For analysis regarding the birthplace effect, we have considered the state and the region (Southeast; Northeast; South; North; and Midwest) of birth. To investigate RAE, the birth date of athletes was distributed into birth quartiles. The first quartile (Q1) comprises the months January, February, and March; the second quartile (Q2) comprises the months April, May, and June; the third quartile (Q3) comprises the months July, August, and September; and the fourth quartile (Q4) comprises the months October, November, and December.

### 2.4. Statistical analysis

Mean and standard deviation (SD) and frequency (%) were used to express the descriptive information. The Chi-square test (χ^2^) was used to present the association between birthplace and RAE between sexes. Similarly, we used the Chi-square test (χ^2^) to verify the association between the birthplace effect and RAE. All analyses were performed considering the total sample and both sexes. These analyses were performed in the SPSS software version 26.0, adopting a significance value of 5%.

## 3. Results

**Figure 1:**
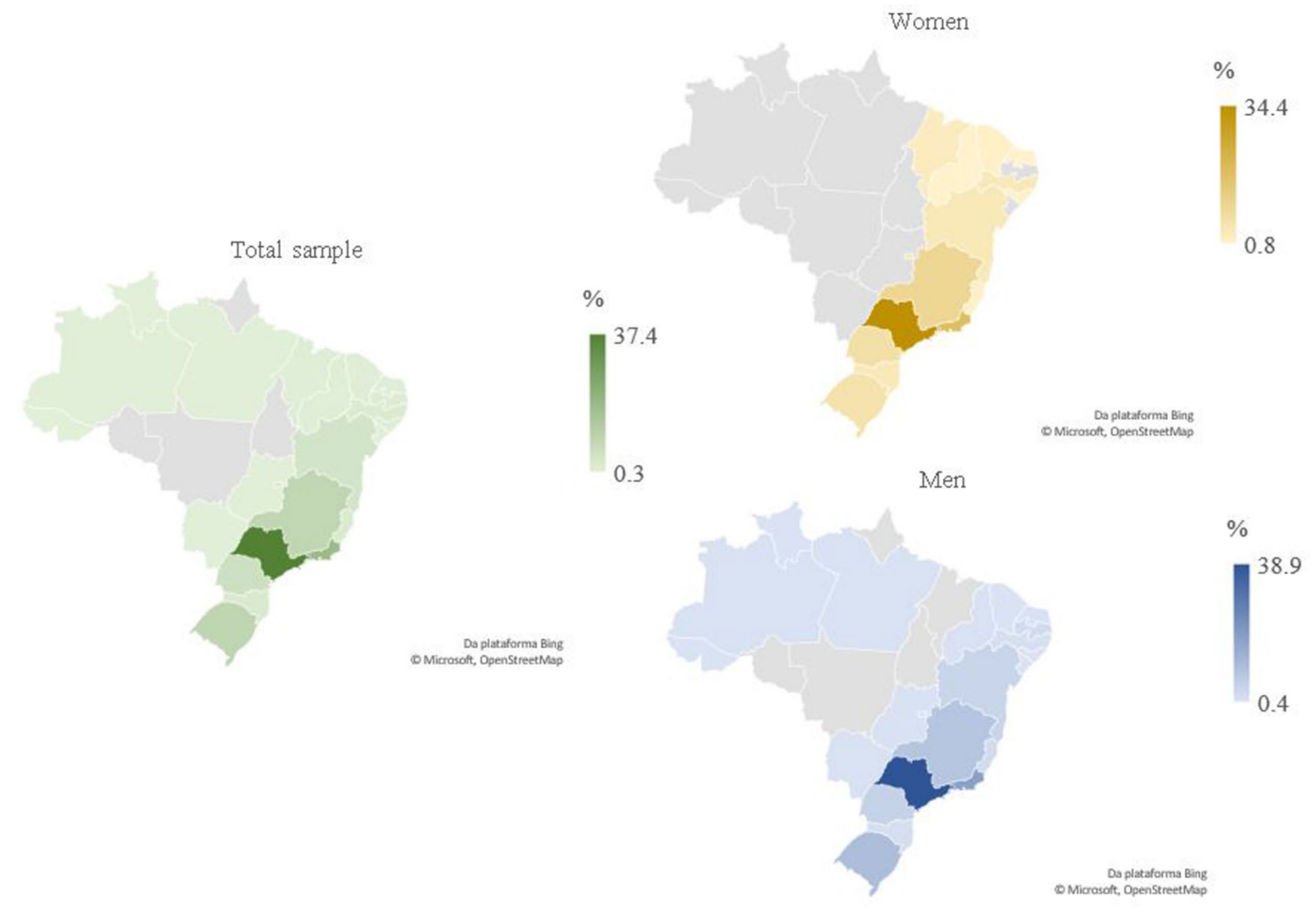
The number of athletes distributed by the state, considering the total sample and both sexes.

The total sample was composed of 388 athletes (woman: 131; men: 257), mean age of 25.5 ± 5.5 years (women: 25.8 ± 5.4 years; men: 25.3 ± 5.6 years), from 23 Brazilian states. Most of the participants competed in team sports, such as soccer (33.5%), volleyball (19.8%), and basketball (10.8%). For both sexes, most of the athletes were from São Paulo state (37.4%), followed by Rio de Janeiro (18.3%), both from the Southeast region (Figure 1). The lowest representativeness was observed for athletes from Acre, Roraima, and Sergipe states. Considering macro-regions of birth, the chi-square test results did not show significant differences between sexes (χ^2^ = 5.69, ρ = 0.128).

**Figure 2:**
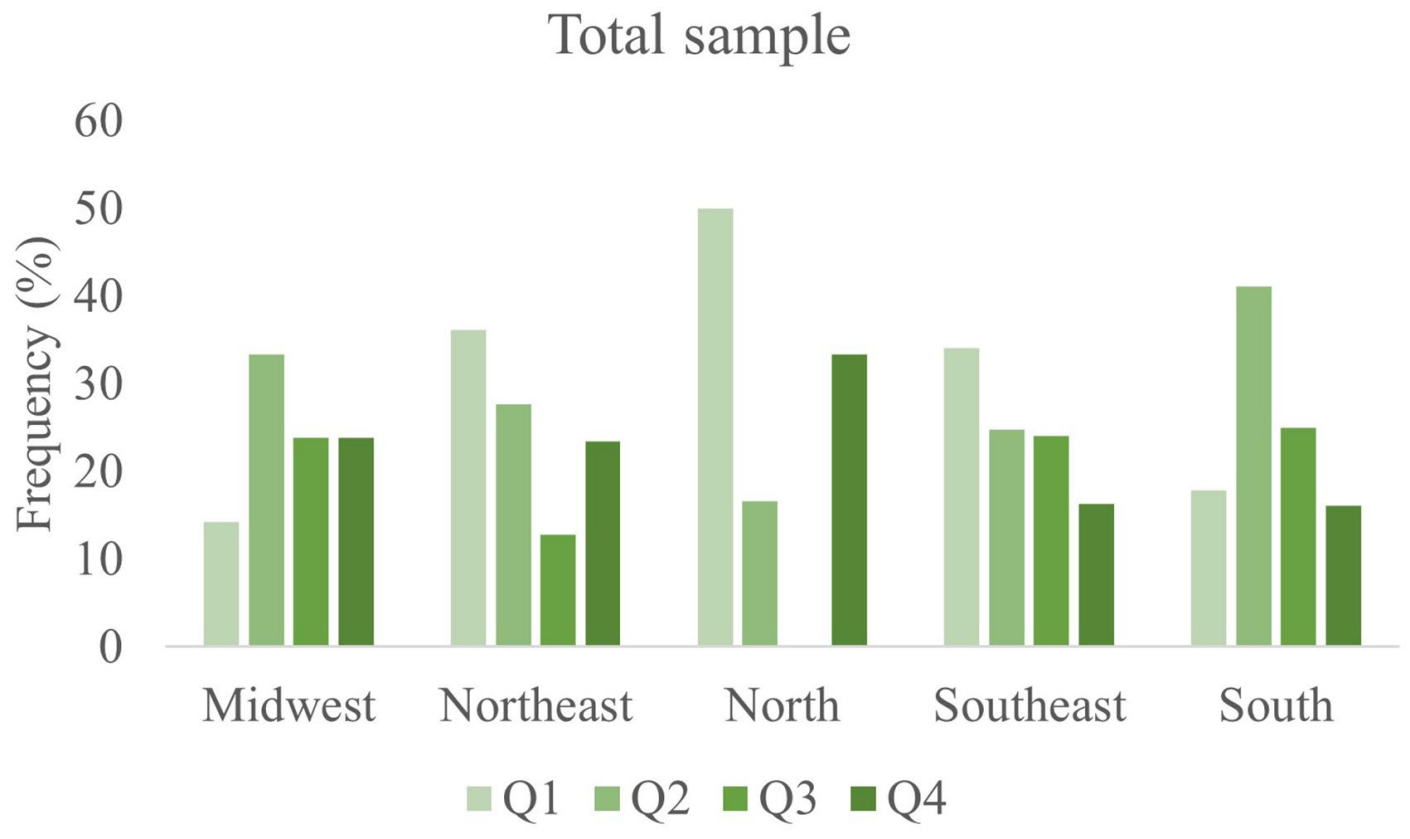
Birthdate distribution according to Brazilian macro-regions.

Regarding the RAE, a higher frequency of athletes was born in the first (31.2%) and second (27.8%) quartiles, while a small frequency was born in the fourth quartile, for both sexes (Figure 2). The birthdate distribution according to birthplace (South; Southeast; North; Northeast; Midwest) showed that the North region presented the highest frequency of athletes born in Q1 (50%), followed by the Northeast and Southeast regions. Most of the athletes from the South were born in Q2 (41.1%), while a similar distribution between the quartiles was found for those from Midwest. The association between birthplace and RAE was tested through the chi-square test. No significant differences for birth quartile and birthplace were verified (χ^2^ = 14.7, value of *p* = 0.09). Similar results were founded for RAE, in which significant differences were not observed between sexes (χ^2^ = 0.530, ρ = 0.912).

## 4. Discussion

The purpose of this study was to verify the existence of birthplace and RAE, as well as the association between birthplace effect and RAE among Brazilian athletes competing in the Olympic Games. The results showed that the highest frequency of athletes was from the Southeast region, especially São Paulo state. These results are following previous studies that investigated Summer Olympic Games (Tozetto et al., 2017; Thuany et al., 2021b), individual sports, such as running and swimming (Gomes-Sentone et al., 2019; Thuany et al., 2021a), soccer (Teoldo and Cardoso, 2021), and public policies programs (Reverdito et al., 2016). Factors explaining these results include demographic characteristics, economic indicators, sports public policies, and financial support (Galatti, 2017). Population and wealth were two of the leading indicators for international countries’ sports success (Bohme and Bastos, 2016). Similarly, the Southeast region has more than half of the Brazilian population and the highest gross domestic product in the country. In addition, these results can be related to the fact that this is the region with the highest concentration of professional teams, sports federations, and hosting major sports events (Junior and Borin, 2017).

A higher frequency of athletes born in the first and second quartile was shown for both sexes, but no significant association was found between regions. RAE was previously demonstrated in basketball, soccer, and ice hockey (Cobley et al., 2009), especially for young athletes. When sports categories are divided according to chronological age, athletes born in the first months (Q1 and Q2) can present an advantage compared to those born in the last months regarding anthropometric and physical fitness variables, for example. A comparison between athletes born in January and December highlighted the differences resulting from the time gap of almost 1 year (physical, morphological, training background differences, and an initial advantage). Those differences can be associated with the higher expectation in January-born athletes as proposed by the Pygmalion effect (Hancock et al., 2013).

In summary, several factors are related to the presence of RAE in sports, such as cognitive, emotional, motivational, and morphological characteristics (Musch and Grondin, 2001). The absence of RAE in the present study can be related to methodological limitations. The statistical analysis was performed on the whole sample and was not stratified by sports modalities. Differences in RAE patterns among Rugby players from different nationalities were previously shown (Kearney, 2017), as well as among Olympic athletes around the world (1896–1996), regardless of sex and type of sports (Joyner et al., 2020).

Although without significant differences, we observed a higher frequency of athletes born in the first semester (Q1 and Q2). Previous studies propose some strategies to mitigate RAE, such as (i) improving selection criteria by changing the dates/ages of cutoff points, (ii) using selection systems based on biological age; (iii) in team sports, holding championships by similar age; and (iv) train scouts so that they can identify and avoid RAE during selection processes (Gil et al., 2021). Although there are many strategies proposed to mitigate this effect, the implementation must be done carefully by testing its possible outcomes, as they can significantly affect the athlete’s life (Webdale et al., 2020). Social issues had already been identified as a decisive factor in this process. Therefore, government agencies, team managers, and coaches also play a crucial role in mitigating these effects (Baker et al., 2010). We did not identify any significant differences for RAE according to sex, although previous research suggested a more significant effect among men, while current studies corroborate our findings (Kelly et al., 2020). Most of the research are conducted in the context of male athletes (Parma and Penna, 2018), but results among female athletes also identified the presence of RAE in different modalities (Smith et al., 2018).

The present study has some limitations. Firstly, we decided to provide a general view of the birthplace effect and RAE among Brazilian Olympic athletes, but differences can be shown considering sports modalities. The differences between states’ characteristics can be associated with sports practices. Secondly, the birthplace can be different from the place where the athlete grew up, but this information is not available to be used. Information about populational birthdate was not considered, since we used data from different years, which impaired the use of 1 year as parameter. In addition, a limited number of data was available to be used in this study. This is an important limitation since important differences can be shown in a more representative sample. However, no previous studies have investigated RAE in the Brazilian regions to the best of our knowledge. The five regions present economic, social, cultural, and demographic differences, influencing sports access and practice, training facilities, and financial support. Investigating the association between the birthplace effect and RAE with sports performance according to the medals won at the Olympics Games (gold, silver, and bronze) or the position in the competition ranking may be a relevant issue for future research, as well as a higher sample size for each branch of sport investigated.

## 5. Conclusion

Most of the Brazilian Olympic athletes were from the Southeast region, especially São Paulo and Rio de Janeiro states. Taking into account the quartiles in which athletes were born, the highest frequency of them were born in Q1 and Q2, and significant differences for RAE according to sex were not identified. Results from the present study can guide public policies in Brazilian regions, especially for the Midwest, North, and Northeast regions, which are underrepresented in Brazilian high-performance sports.

## Data availability statement

The raw data supporting the conclusions of this article will be made available by the authors, without undue reservation.

## Ethics statement

Ethical review and approval was not required for the study on human participants in accordance with the local legislation and institutional requirements. Written informed consent from the patients/ participants or patients/participants legal guardian/next of kin was not required to participate in this study in accordance with the national legislation and the institutional requirements.

## Author contributions

MT: conceptualization and formal analysis. MT, ML, JC, and TA: methodology. MT and DV: writing—original draft preparation. PN, BK, KW, and TG: writing—review and editing. All authors contributed to the article and approved the submitted version.

## Conflict of interest

The authors declare that the research was conducted in the absence of any commercial or financial relationships that could be construed as a potential conflict of interest.

## Publisher’s note

All claims expressed in this article are solely those of the authors and do not necessarily represent those of their affiliated organizations, or those of the publisher, the editors and the reviewers. Any product that may be evaluated in this article, or claim that may be made by its manufacturer, is not guaranteed or endorsed by the publisher.

## Footnotes

^1^ www.cob.org.br/pt/cob/time-brasil/atletas/
